# Makeup increases attractiveness in male faces

**DOI:** 10.1371/journal.pone.0275662

**Published:** 2022-11-03

**Authors:** Carlota Batres, Hannah Robinson

**Affiliations:** Department of Psychology, Franklin and Marshall College, Lancaster, PA, United States of America; St John’s University, UNITED STATES

## Abstract

Makeup is commonly attributed with increasing attractiveness in female faces, but this effect has not been investigated in male faces. We therefore sought to examine whether the positive effect of makeup on attractiveness can be extended to male faces. Twenty men were photographed facing forward, under constant camera and lighting conditions, with neutral expressions, and closed mouths. Each man was photographed twice: once without any cosmetics applied and another time with subtle cosmetics applied by a professional makeup artist. Two hundred participants then rated those 40 images on attractiveness. The male faces were rated as higher in attractiveness when presented wearing makeup, compared to when presented not wearing makeup. This was true for both male and female raters, and whether analyzing the data using a by-participant or a by-face analysis. These results provide the first empirical evidence that makeup increases attractiveness in male faces. Following work on female faces, future research should examine the effect of makeup on several other traits in male faces. The market for male cosmetics products is growing and evolving and this study serves as an initial step in understanding the effect of makeup on the perceptions of male faces.

## Introduction

Much research has been devoted to understanding the effect of makeup on perceptions. Several studies using carefully controlled before and after photographs have found that women’s faces with makeup are judged as more attractive by both male and female raters than the same faces without makeup [1–3]. This effect has been found across several styles of makeup, across ethnicities, and across various ages [4]. Moreover, this effect has been found to influence real-world outcomes, such as tipping behaviors and jury decisions [5, 6].

Makeup is believed to increase attractiveness through its effects on visual features, such as skin homogeneity [7] and facial contrast [8]. Makeup has been found to increase skin evenness, using both perceptual and physical measures [7], and skin homogeneity has been found to increase attractiveness [9]. Makeup has also been found to increase facial contrast (i.e., the difference in coloration between facial features and the surrounding skin) in female faces, which also makes faces appear more attractive [8].

This effect of makeup on attractiveness is well documented for women [1, 10–14], but no study has investigated it in men. The market for male cosmetics products is growing and evolving [15]. This includes the purchase of creams, lotions, face scrubs, and so forth [16]. In terms of makeup, certain products that have commonly only been used by women are starting to be marketed for men. For instance, several companies now sell concealers, powders, bronzers, and eyebrow gels specifically for men [17]. In the past few years, purchases of these products by men have been steadily increasing [17]. The positive effect of these products on perceptions of attractiveness has been studied empirically in female faces, but not in male faces.

Given the effects of attractiveness on real-world outcomes (e.g., election results [18], sentencing decisions [19]), we sought to examine whether the positive effect of makeup can be extended to male faces. Previous studies have found that skin homogeneity increases perceptions of attractiveness in male facial skin [20]. Previous research has also found that lower facial contrast makes faces appear more masculine [8]. Additionally, bone structure in men (e.g., facial width-to-height ratio) affects perceptions of attractiveness [21]. Therefore, we predicted that male faces with makeup would be rated as more attractive than male faces without makeup, since makeup could potentially be applied to influence skin homogeneity, facial contrast, and bone structure perceptions.

## Materials and methods

### Stimuli

Ethical approval was received from the Franklin and Marshall College Institutional Review Board. Participants were recruited through word-of-mouth, social media, and paper flyers. Upon arrival at the lab, written informed consent was obtained from all the participants allowing the use of their photographs for research purposes. Twenty men ranging in ages from 18 to 36 (*M*_age_ = 21.90, *SD* = 4.01) were photographed facing forward, under constant camera and lighting conditions, with neutral expressions, and closed mouths. Nineteen of the men self-reported being White and one man self-reported being Asian.

**Fig 1:**
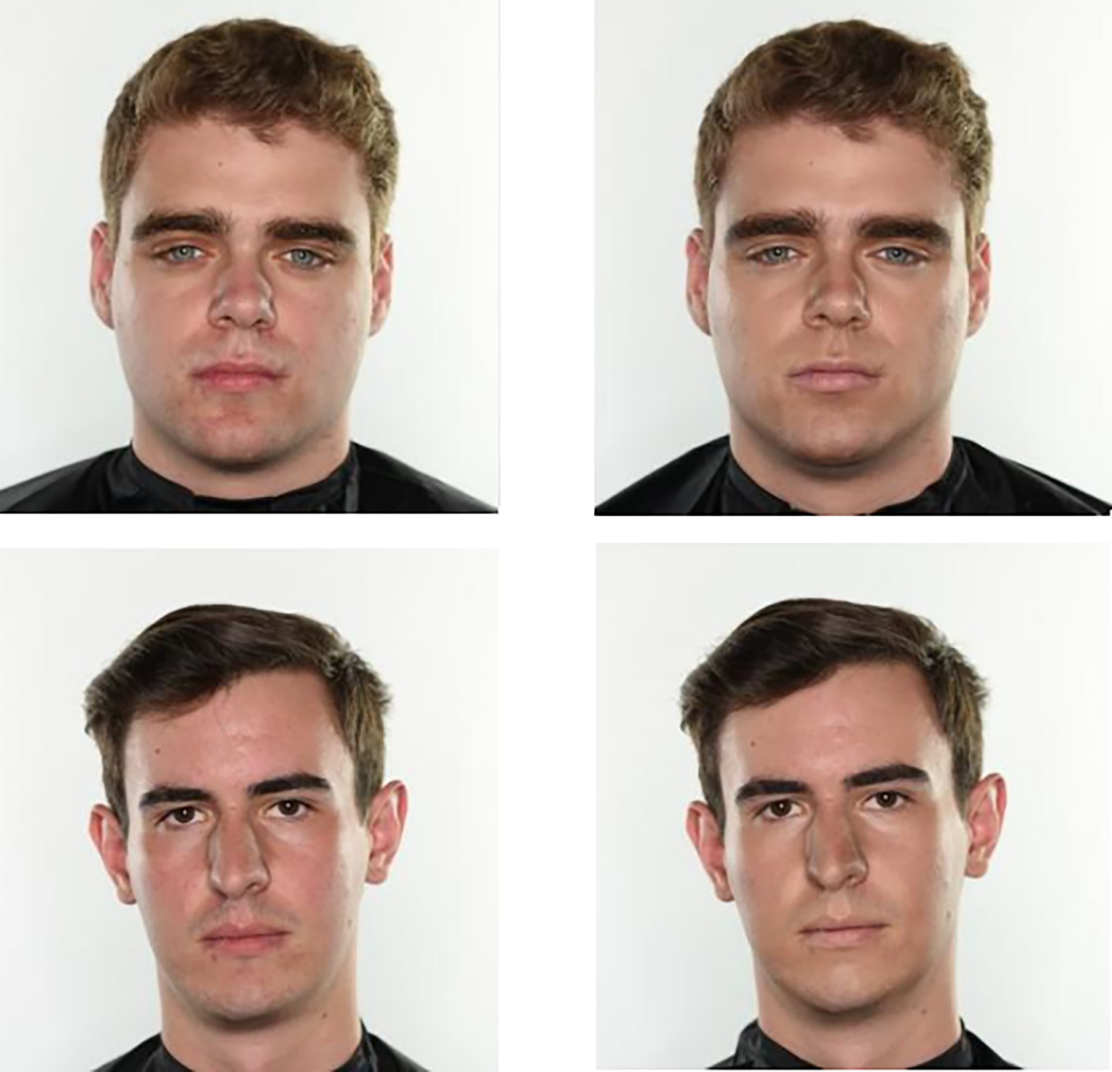
Example stimuli. Two of the participants without cosmetics (left) and with cosmetics applied by a professional makeup artist (right).

Each man was photographed two times: one time without any cosmetics applied and a second time with cosmetics applied by a professional makeup artist. The professional makeup artist applied a range of cosmetics on the participants. For instance, concealer, powder, etc. The professional makeup artist was instructed to increase skin homogeneity, decrease facial contrast, and accentuate the bone structure without it being too obvious that the targets were wearing makeup. This resulted in a total of 40 images, where each of the 20 male faces had a no makeup image and a makeup image (Fig 1). Participants were also asked to complete a questionnaire that requested their demographic information (e.g., sex, age). Upon completion, participants were compensated $10 for taking part in the study.

### Participants and procedures

Ethical approval was received from the Franklin and Marshall College Institutional Review Board. Participants were recruited online through Amazon’s Mechanical Turk platform and each participant provided informed consent. Two hundred participants completed the survey through the platform (*M*_age_ = 39.71, *SD* = 12.56). One hundred and twenty four of the participants identified as male and 76 identified as female. All participants were located in the United States.

Participants were instructed that they would be viewing several faces for which they would have to rate attractiveness. More specifically, participants were asked “How attractive is this face?”, where 1 = “Not at all attractive” and 7 = “Very attractive”. The no makeup and makeup conditions were intermixed and participants rated both versions of each face individually (i.e., all 40 images) in random order, with no time limit. Participants were also asked for demographic information (e.g., sex, age). Upon completion, participants were paid $0.80 for participation in the study.

## Results

**Fig 2:**
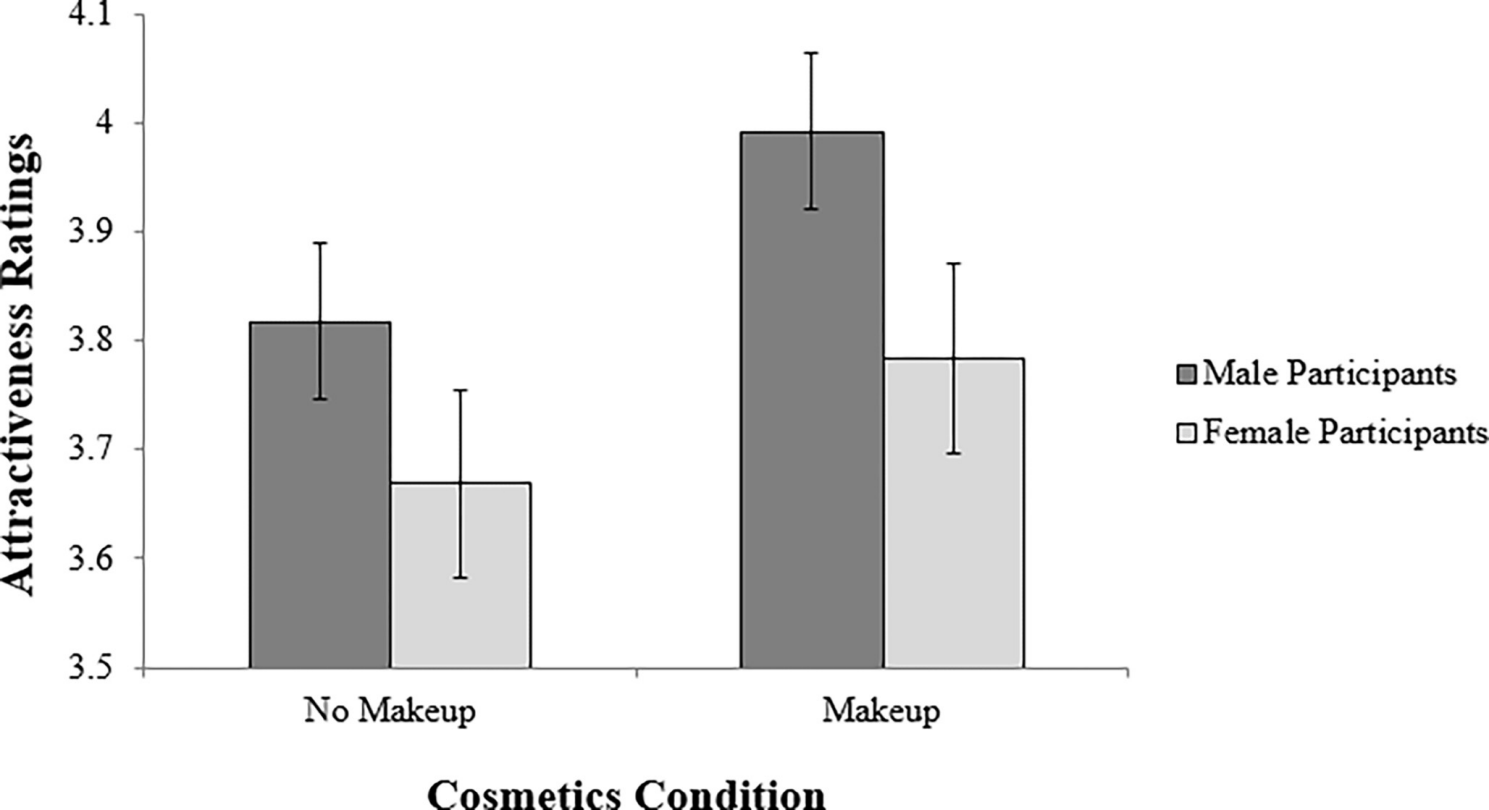
Ratings of attractiveness depending on makeup condition. Comparison of attractiveness ratings for male faces with no cosmetics and with subtle cosmetics applied by a professional makeup artist. Error bars indicate the standard error of the mean.

Participants showed high inter-rater reliability for the ratings of attractiveness (Cronbach’s α = 0.95). When conducting a by-participant analysis, there was a statistically significant difference between the faces with makeup and the faces without makeup (*t* (199) = 6.35, *p* < 0.001, Cohen’s *d* = 0.34). More specifically, the faces with makeup were rated as being more attractive (*M* = 3.91, *SD* = 0.79) than the faces without makeup (*M* = 3.76, *SD* = 0.78). When splitting by participant sex, both men (*t* (123) = 6.06, *p* < 0.001, Cohen’s *d* = 0.32) and women (*t* (75) = 2.74, *p* < 0.01, Cohen’s *d* = 0.36) rated the faces with makeup as significantly more attractive than the faces without makeup (Fig 2).

When conducting a by-face analysis, the difference between the makeup and the no makeup conditions was also statistically significant (*t* (19) = 2.95, *p* < 0.01, Cohen’s *d* = 0.23). Out of the 20 faces, four were rated as less attractive with makeup and 16 were rated as more attractive with makeup.

## Discussion

Evidence from a perceptual study supported the hypothesis that subtle cosmetics would make male faces look more attractive. We found that the same faces were rated as more attractive when they were wearing makeup, compared to when they were not wearing makeup. This effect is in line with previous research done with female faces [1–3].

While the difference between men with and without makeup was statistically significant, the effect size was small. This is in contrast with the research done with female faces which has found a large effect of makeup on attractiveness (e.g., η^2^ = 0.33 [22]). The small effect we found in male faces is probably due to the fact that we wanted the makeup to appear natural so as to not activate any stereotypes participants may have about male makeup [23]. Regardless, given the effects of attractiveness on real-world outcomes [5, 6], even a small effect can have large consequences.

One point to note from our study is that not all of the faces were found to be more attractive with makeup. Previous research has found that identity has an effect size that is 1.36 times larger than the effect size attributed to makeup [22]. In our study, four out of the 20 faces were not rated as more attractive with makeup and it would be interesting for future research to investigate which types of faces gain the most from makeup applications. Additionally, future research is also needed to investigate what type of makeup increases male attractiveness. In this study, the professional makeup artist used a range of cosmetics on the participants (e.g., concealer, powder) and it would be interesting to limit applications in order to investigate the individual effects of these products.

It would also be interesting to further examine what aspect of the makeup application most greatly influences attractiveness perceptions. In our study, the professional makeup artist was instructed to increase skin homogeneity, decrease facial contrast, and accentuate the bone structure. While we got an overall effect of makeup, we are not able to dissociate which of these factors was the most important. For example, perhaps skin homogeneity is responsible for the entire positive effect, or maybe it is a combination of all three factors.

Lastly, this study only looked at perceptions of attractiveness in male faces. However, there is vast amount of research examining the effects of makeup on several other traits in female faces. For instance, likeability [4], leadership ability [24], trustworthiness [25], confidence [26], earning potential [26], and competence [25]. It would therefore be interesting to also examine the effect of makeup on these traits in male faces.

### Conclusions

In conclusion, several studies have found that makeup increases attractiveness in female faces [1–3]. Here, we present the first empirical evidence that this effect extends to male faces. More specifically, we found that the same male faces were rated as more attractive when presented wearing makeup, compared to when presented not wearing makeup. This study thus serves as an initial step in understanding the effect of makeup on the perceptions of male faces.
